# Effect of digoxin on all-cause and cardiovascular mortality in patients with atrial fibrillation with and without heart failure: an umbrella review of systematic reviews and 12 meta-analyses

**DOI:** 10.1007/s00228-023-03470-y

**Published:** 2023-03-06

**Authors:** Gianluca Gazzaniga, Danilo Menichelli, Francesco Scaglione, Alessio Farcomeni, Arianna Pani, Daniele Pastori

**Affiliations:** 1 Department of Medical Biotechnology and Translational Medicine, Postgraduate School of Clinical Pharmacology and Toxicology, Università degli Studi di Milano, 20122 Milan, Italy; 2 Department of General and Specialized Surgery “Paride Stefanini”, Sapienza University of Rome, 00185 Rome, Italy; 3 Department of Oncology and Hemato-Oncology, Università degli Studi di Milano, 20122 Milan, Italy; 4 Department of Chemical-Clinical and Microbiological Analyses, Grande Ospedale Metropolitano Niguarda, 20162 Milan, Italy; 5 Department of Economics and Finance, University of Rome “:Tor Vergata”, 00133 Rome, Italy; 6 Department of Clinical, Internal, Anesthesiological, and Cardiovascular Sciences, Sapienza University of Rome, 00185 Rome, Italy

**Keywords:** Digoxin, All-cause mortality, Cardiovascular mortality, Atrial fibrillation, Heart failure

## Abstract

**Purpose:**

To perform a systematic umbrella review with meta-analysis to evaluate the certainty of evidence on mortality risk associated with digoxin use in patients with atrial fibrillation (AF) with or without heart failure (HF).

**Methods:**

We systematically searched MEDLINE, Embase, and Web of Science databases from inception to 19 October 2021. We included systematic reviews and meta-analyses of observational studies investigating digoxin effects on mortality of adult patients with AF and/or HF. The primary outcome was all-cause mortality; secondary outcome was cardiovascular mortality. Certainty of evidence was evaluated by the Grading of Recommendations Assessment, Development and Evaluation (GRADE) tool and the quality of systematic reviews/meta-analyses by the A MeaSurement Tool to Assess systematic Reviews 2 (AMSTAR2) tool.

**Results:**

Eleven studies accounting for 12 meta-analyses were included with a total of 4,586,515 patients. AMSTAR2 analysis showed a high quality in 1, moderate in 5, low in 2, and critically low in 3 studies. Digoxin was associated with an increased all-cause mortality (hazard ratio [HR] 1.19, 95% confidence interval [95%CI] 1.14–1.25) with moderate certainty of evidence and with an increased cardiovascular mortality (HR 1.19, 95%CI 1.06–1.33) with moderate certainty of evidence. Subgroup analysis showed that digoxin was associated with all-cause mortality both in patients with AF alone (HR 1.23, 95%CI 1.19–1.28) and in those with AF and HF (HR 1.14, 95%CI 1.12–1.16).

**Conclusion:**

Data from this umbrella review suggests that digoxin use is associated with a moderate increased risk of all-cause and cardiovascular mortality in AF patients regardless of the presence of HF.

**Trial registration:**

This review was registered in PROSPERO (CRD42022325321).

**Supplementary Information:**

The online version contains supplementary material available at 10.1007/s00228-023-03470-y.

## Introduction

The management of patients suffering from atrial fibrillation (AF) is multifactorial including thromboprophylaxis for cardioembolic stroke by anticoagulant treatment, symptoms management, and rate and rhythm control by anti-arrhythmic drugs [1, 2]. Indeed, beyond anticoagulation therapy, rhythm and rate control strategies are cornerstone for the acute and chronic management of patients with AF [3]. Among anti-arrhythmic drugs, digoxin is a still widely used drug to control heart rate in AF patients. The 2020 guidelines from the European Society of Cardiology (ESC) recommend beta-blockers and/or digoxin to control heart rate in AF patients with left ventricular ejection fraction < 40% (class I level of evidence B) [3]. In addition, the ESC guidelines recommend the long-term use of digoxin in patients in whom an adequate rate control cannot be achieved by beta blockers at maximum tolerated dose or when beta-blockers are contraindicated or not tolerated with low class of evidence (IIa) [3].

Digoxin is also recommended by the “2021 ESC guidelines for the diagnosis and treatment of acute and chronic heart failure” for the treatment of patients with heart failure (HF) and sinus rhythm to reduce the risk of hospitalization and symptoms burden (class IIb level B) [4]. A similar recommendation is provided by the 2022 AHA/ACC/HFSA Guideline for the Management of Heart Failure [5].

During the last decades, concerns regarding the safety of digoxin have been raised. In particular, some studies showed an increased risk of death in patients with AF treated with digoxin, especially when supratherapeutic blood concentrations are reached [6, 7]. Given the possibility of the presence of an indication bias (i.e., administration of digoxin to sicker patients), also propensity-matched studies have been performed providing divergent conclusions [8, 9].

However, several systematic reviews and meta-analyses investigating the association of digoxin with all-cause and cardiovascular mortality reported conflicting evidence [10, 11].

Given the impossibility of obtaining data from a randomized trial testing the safety and efficacy of digoxin in addition to standard therapy, we performed an umbrella review of systematic reviews and meta-analyses, focusing on patients with AF with and without HF, in whom the use of digoxin has recommendation by international guidelines.

## Methods

This review was registered in PROSPERO (CRD42022325321). This umbrella review was conducted in accordance with the Preferred Reporting Items for Systematic Reviews and Meta-Analyses (PRISMA) guidelines [12]. (Supplementary Fig. 1).

### Data sources and searches

A systematic literature search was conducted by two independent authors on MEDLINE, Embase, and Web of Science databases from inception to 19 October 2021. Keywords used to perform the search were “digoxin”, “atrial fibrillation”, “mortality”, and “meta-analysis” combined with Boolean operators were used to find articles. Search strategy was adapted for each database; a complete list of search strings is available in Supplementary Material 1.

### Study selection

Criteria of inclusion were defined as follows: Meta-analysis of studies investigating digoxin effects compared to standard of care on mortality of patients with AF; patients included in studies must be at least 18 years old; effect sizes must be provided as relative risk (RR) or hazard ratio (HR) with 95% confidence intervals (95%CI). Only articles with full-text available were considered. No restrictions were placed on language or publication date. Meta-analyses which did not report data concerning mortality were excluded. Multiple meta-analyses reported in a single paper (e.g., multiple outcomes or based on different types of studies) were included separately.

Study selection was performed by two independent authors, and disagreements were resolved through discussion with the senior author. Titles and abstracts of each article were screened to remove duplicates, and full texts of promising articles were read to assess eligibility. Reference lists of eligible articles were hand-searched to identify additional relevant meta-analyses.

### Data extraction

Two investigators independently extracted the following data from each eligible study: name of first author; year of publication; outcomes; databases whose searches were based on; period of time searched; number and type of included studies; follow-up period; digoxin indication; number of patients with AF; overall mortality; cardiovascular mortality; mortality in patients with only AF; and mortality in patients with AF and HF.

### Outcomes

All-cause mortality was the primary outcome. Secondary endpoint was cardiovascular mortality. A subgroup analysis in patients with AF alone or AF and HF was performed.

### Quality evaluation and risk of bias assessment

Quality of each included meta-analysis was evaluated with the assessment of multiple systematic reviews (AMSTAR) 2 tool [13]. This tool aims at evaluating systematic reviews quality by answering “no”, “partial yes”, or “yes” to 16 different items. Items 4, 9, 11, 12, and 15 are considered critical domains. The quality of studies was defined as follows: high (no or 1 non-critical weakness), moderate (more than 1 non-critical weakness), low (1 critical flaw with or without non-critical weaknesses), or critically low (more than one critical flaw with or without non-critical weaknesses) (Table 1).

**Table 1:** AMSTAR2 tool evaluation of the quality of the included systematic reviews/meta-analyses

**Author**	**Q1**	**Q2**	**Q3**	**Q4**	**Q5**	**Q6**	**Q7**	**Q8**	**Q9**	**Q10**	**Q11**	**Q12**	Q**13**	**Q14**	**Q15**	**Q16**	Overall quality
**Bavishi et al. **[25]	Yes	No	No	Partial Yes	No	No	No	Partial Yes	Partial Yes	No	Yes	Yes	No	No	Yes	Yes	Moderate
**Chamaria et al.** [26]	Yes	No	No	Partial Yes	Yes	Yes	No	Yes	Yes	No	Yes	Yes	No	Yes	Yes	Yes	Moderate
**Ouyang et al.** [10]	Yes	No	No	Partial Yes	Yes	Yes	No	Partial Yes	Yes	No	Yes	Yes	No	Yes	Yes	Yes	Moderate
**Wang et al.** [27]	Yes	No	No	Partial Yes	Yes	No	No	Partial Yes	No	No	No	No	No	Yes	Yes	Yes	Critically low
**Chen et al.** [28]	Yes	No	No	Partial Yes	Yes	Yes	No	Partial Yes	No	No	Yes	No	No	Yes	Yes	Yes	Critically low
**Ziff et al.** [11]	Yes	Yes	Yes	Yes	No	Yes	No	Yes	Yes	No	Yes	Yes	Yes	Yes	Yes	Yes	Moderate
**Qureshi et al.** [29]	Yes	No	No	Partial Yes	Yes	No	No	Partial Yes	No	No	Yes	No	Yes	Yes	Yes	Yes	Critically low
**Zeng et al.** [30]	Yes	No	No	Partial Yes	No	Yes	No	Partial Yes	Yes	No	Yes	Yes	Yes	Yes	Yes	Yes	Moderate
**Sethi et al.** [31]	Yes	Yes	Yes	Yes	Yes	Yes	No	Yes	Yes	Yes	Yes	Yes	Yes	Yes	Yes	Yes	High
**Vamos et al.** [32]	Yes	No	No	Partial Yes	Yes	No	No	Yes	Yes	No	Yes	No	No	No	Yes	Yes	Low
**Wang et al.** [33]	Yes	Yes	No	Yes	Yes	Yes	No	Yes	Yes	No	Yes	No	No	No	Yes	Yes	Low

### Data synthesis and analysis

Meta-analyses for each endpoint separately were performed based on random effects, using the logarithm of hazard ratios (HRs) associated as outcome. Inverse variance weights were used in all cases. Pooled effects were obtained through maximum likelihood.

Heterogeneity was evaluated by calculating the *I*^2^ index. According to arbitrary cut-offs, low, moderate, and high heterogeneity was defined as an *I*^2^ of < 25%, 25–75%, and > 75%, respectively.

Publication bias was assessed for studies reporting outcomes according to digoxin use, with the use of funnel plots. Egger’s test was also performed.

Analyses were performed using the R (R Development Core Team) software version 3.6.1; statistical significance level was set at 0.05, and all *p* values were two-tailed.

### Certainty of evidence

Two authors independently evaluated certainty of evidence using GRADEproGDT according to the GRADE Handbook [14]. GRADE categorizes certainty of evidence into very low, low, moderate, or high; the higher the category, the greater the confidence that the true effect is close to the reported findings. The following characteristics are considered in order to assess the right category: design of study (observational, randomized clinical trial), inconsistency across studies (*I*^2^ statistics), imprecision of the findings, indirectness (e.g., due to mixed outcome), publication bias, size effect, and presence of dose–response gradient.

## Ethics approval

This is an umbrella review with meta-analysis. No ethical approval is required.

## Results

Three hundred ninety-seven articles were obtained from the initial search. After duplicates removal, 350 papers were evaluated. After a first screening, 39 studies were eligible for detailed analysis, but 5 had no full-text available. Finally, 11 studies met the eligibility criteria and were included in the umbrella review. The strategy search is summarized in PRISMA flow diagram (Supplementary Fig. 1).

Table 1 shows AMSTAR 2 items evaluation for every included meta-analysis. Overall, out of the 11 papers, 1 had a high, 5 moderate, 2 low, and 3 critically low quality. In particular, as far as it regards critical domains, each paper reported a proper use of comprehensive literature search queries and a significant publication bias assessment (Q4, 11/11, and Q15, 11/11, respectively). Almost every paper used an appropriate method for statistical combination of the results (Q11, 10/11), while some critical issues were found in the evaluation of technique for assessing risk of bias and in determining its implication on the results of the meta-analysis (Q9 8/11 and Q12 6/11, respectively).

Strategy search, year of publication, inclusion, and exclusion criteria are reported in Table 2.

**Table 2:** Strategy search, inclusion, and exclusion criteria used in each systematic reviews/meta-analysis

**Author**	**Year**	**Database searched**	**Time limit for strategy search**	**Inclusion criteria**
**Bavishi et al.** [25]	2015	PubMed, Scopus, Cochrane collaboration central register of controlled trials, Embase	February 2014	- Evaluation of long-term all-cause mortality with the use of digoxin in patients with HF and AF -FU ≥ 6 months
**Chamaria et al.** [26]	2015	Medline, WOS	2015	- Prospective or retrospective observational studies with a primary objective to analyze the association between digoxin and all-cause mortality in patients with AF with or without HF - Digoxin compared to no digoxin or any other rate-controlling drug in pts with AF - FU ≥ 6 months - Adjusted HR was reported - All-cause mortality was the endpoint
**Ouyang et al.** [10]	2015	PubMed, Embase	29/12/2014	- Human research - Prospective or retrospective studies assessing the association between digoxin use and risk for all-cause mortality in AF patients - Follow-up > 1 year - Described adjustment for potential confounding - Reported effect estimates with CIs, standard errors, or sufficient information to calculate these
**Wang et al.** [27]	2015	PubMed, Embase, and the Cochrane central databases	1/12/2014	1) Clinical studies comparing the risk of all-cause mortality in those individuals that received or did not receive digoxin 2) Paroxysmal or persistent patients with AF
**Chen et al.** [28]	2015	PubMed, Embase, and the Cochrane library	31/08/2015	Aged 18 years; adjusted RR and 95%CIs for all-cause mortality associated with digoxin treatment; FU > 1 year
**Ziff et al.** [11]	2015	Medline, Embase, the Cochrane library, reference lists, and ongoing studies	July 2014	- Comparative outcomes with digoxin and control
**Qureshi et al.** [29]	2016	MEDLINE, Embase, Google Scholar, Web of Science	15/12/2014	- Observational studies and analyses from clinical trials
**Zeng et al.** [30]	2016	PubMed, Embase	31/07/2015	(1) The study designs were cohort studies, case–control studies or RCTs (2) The outcome of interest was death from any cause, cardiovascular death, arrhythmic death or stroke (3) RR and the corresponding 95%CI were reported (4) Studies were independent
**Sethi et al.** [31]	2018	CENTRAL, MEDLINE, Embase, LILACS, SCI-Expanded, BIOSIS	October 2016	-Randomized clinical trials comparing digoxin versus placebo, no intervention, or other medical interventions in patients with AF or atrial flutter
**Vamos et al.** [32]	2019	Medline, Cochrane	March 2018	- Full-size articles in English - AF or HF population - Report of adjusted results of effects of digoxin on all-cause mortality - Effect sizes provided as HR with 95%CI
**Wang et al.** [33]	2021	Pubmed, Embase, Cochrane library	September 2020	- FU > 6 months - RCT, observational, retrospective analysis -Digoxin compared with no digoxin or other heart rate control treatment in patients with AF - HRs with 95%CIs for outcomes associated with digoxin treatment were reported - Length of FU ≥ 6 months - All-cause mortality was the endpoint

*AF* atrial fibrillation, *CI* confidence intervals, *FU* follow-up, *HF* heart failure, *HR* hazard ratios, *RCT* randomized controlled trials, *RR* relative risks

Table 3 summarizes meta-analysis characteristics. Each study reported synthesis of results expressed as HR or RR. One paper (Sethi et al.) included only RCTs, while the others included observational studies and data from registries as well. A total of 4,586,515 patients were included. The length of follow-up ranged from 0.4 to 4.7 years. Notably, Ziff and colleagues [11] reported data on all-cause mortality and cardiovascular mortality separately for observational and interventional studies; given the different populations in the two analyses, we considered them as two different studies.

**Table 3:** Outcomes and number of patients treated or not with digoxin

**Author**	**Outcomes**	**Studies included**	**Type of studies included**	**Total number of patients included**	**FU**	**Digoxin indications**	**Number of included patients with AF**	***N*** **patients receiving Digoxin**	***N*** **patients not receiving digoxin (control)**	**Measure of effect size**
**Bavishi et al.** [25]	All-cause mortality in patients with HF and AF	10	4 data from registries 4 single-centers cohort 2 post-hoc analysis of RCT	76,100	> 6 months	AF + HF	76,100	NS	NS	RR
**Chamaria et al.** [26]	Mortality with digoxin use in patients with AF and HF	12	Observational (6 prospective and 6 retrospective)	321,944	0.8 y min–4.6 years max	AF, AF + HF	321,944	83,630	238,314	HR
**Ouyang et al.** [10]	Mortality risk in AF patients treated with digoxin	11	Observational (4 prospective and 7 retrospective)	318,191	1–4.6 years	AF, AF + HF	318,191	87,834	229,971	HR
**Wang et al.** [27]	Mortality risk in AF patients treated with digoxin	8	Prospective and retrospective cohort	302,738	From 1.2 to 4.6 years	AF, AF + HF	302,738	76,085	226,653	HR
**Chen et al.** [28]	Primary: all-cause mortality associated with digoxin Secondary: mortality according to heart function status	17	Observational (7 prospective and 5 retrospective cohort), 5 post-hoc analysis of RCT	408,660	> 1 year	AF, AF + HF	408,660	109,719	298,941	RR
**Ziff et al.** [11]	Primary: all-cause mortality Secondary: mortality, all cause hospital admissions, CV hospital admissions, and HF hospital admissions	52 in systematic review 41 in meta-analysis	26 observational, 9 post-hoc analysis of RCT 7 RCTs	621,845	3.7 years (SD 2.4)	AF + HF	46,274	144,593	476,984	HR/RR
**Qureshi et al.** [29]	Primary: all-cause mortality Secondary: CV mortality	19	9 post-hoc analyses of RCT, 10 observational cohorts	501,681	0,4–4,7 years	AF, AF + HF	501,681	111,978	389,643	HR
**Zeng et al.** [30]	Death for any cause, CV death, arrhythmic death or stroke	22	Observational (11 retrospective and 6 prospective cohorts) and 5 post-hoc analysis of RCT	586,594	From 6.0 to 56.4 months	AF, AF + HF	573,114	NS	NS	RR
**Sethi et al.** [31]	Primary: all-cause mortality, SAE (as defined by ICH guidelines), QoL Secondary outcomes: heart failure, stroke, heart rate control, and conversion to sinus rhythm	32 (28 RCT)	RCT	2223	1 h–24 weeks	AF, AF + HF	522	268	254	RR
**Vamos et al.** [32]	Mortality in AF and in HF	37 studies (23 AF)	Observational (11 retrospective and 5 prospective), 7 post-hoc analysis of RCTs	825,061	6 months (?)	AF, HF	627,620	130,616	497,004	HR
**Wang et al.** [33]	All-cause mortality, all-cause hospitalization and SAE, including SEE/stroke, MI, CV mortality, non-CV mortality, and SCD	29	8 post-hoc analysis of RCT; 21 observational cohort studies (10 retrospective and 11 prospective)	621,478	> 6 months	AF, AF + HF	621,478	NS	NS	HR

*AF* atrial fibrillation, *CV* cardiovascular, *FU* follow-up, *HF* heart failure, *NS* not specified, *RCT* randomized clinical trial, *SAE* serious adverse event, *HR* hazard ratio, *RR* relative risk

Funnel plots reported in the Supplementary Fig. 2 did not show significant publication bias.

### All-cause and cardiovascular mortality

**Fig. 1:**
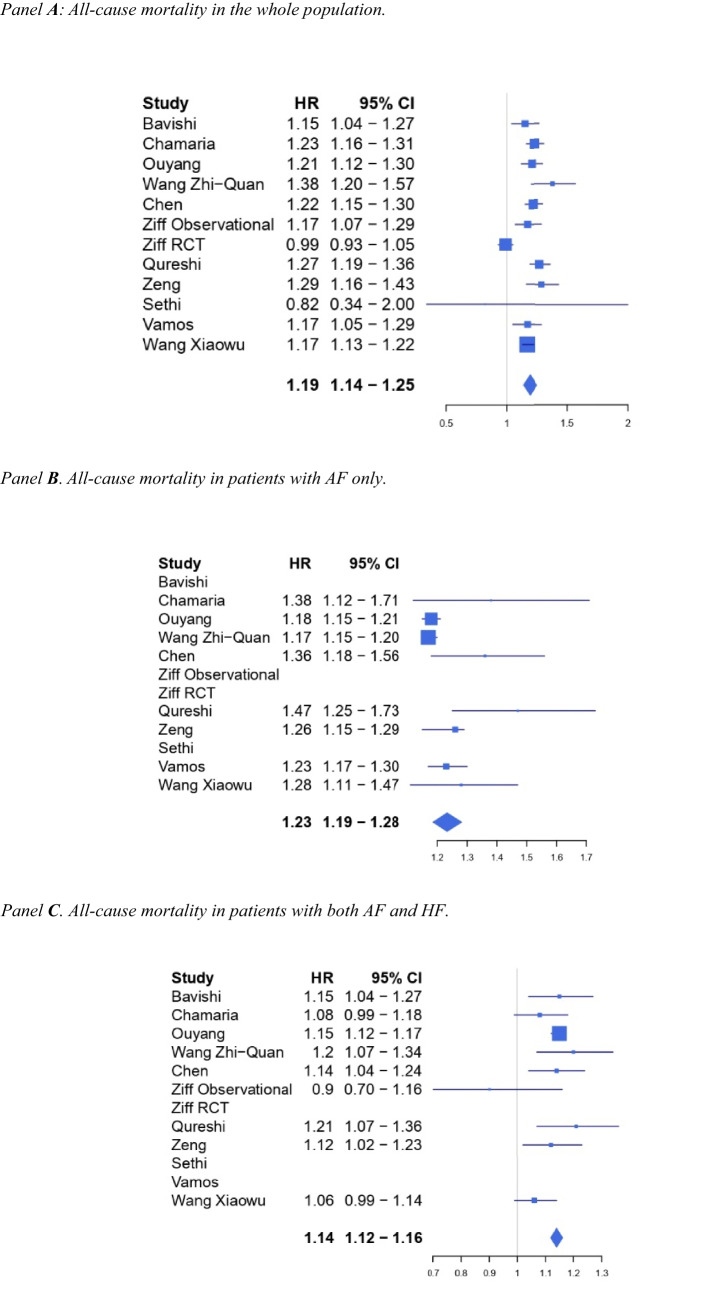
Forest plot for all-cause mortality. **A** Overall. **B** AF only. **C** AF + HF

All the included studies reported data on the primary outcome, the all-cause mortality. Figure 1 shows the results of our umbrella review concerning mortality. Digoxin was associated with an increased mortality in the overall population (HR 1.19, 95%CI 1.14–1.25, panel A) with moderate certainty of evidence according to the GRADE (Table 4) and moderate-high heterogeneity (I^2^ 75.8%).

**Table 4:** GRADE tool

**Outcome** **№ of participants (studies)**	**Relative effect (95%CI)**	**Certainty**	**Comments**
All-cause mortality (12 meta-analyses)	HR 1.19 (1.14 to 1.25)	⨁⨁⨁◯ Moderate	The evidence suggests that digoxin results in an increase in mortality
Cardiovascular mortality (5 meta-analyses)	HR 1.19 (1.06 to 1.33)	⨁⨁⨁◯ Moderate	The evidence suggests that digoxin results in an increase in cardiovascular mortality
Mortality in patients with atrial fibrillation only (8 meta-analyses)	HR 1.23 (1.19 to 1.28)	⨁⨁◯◯ Low^a^	Digoxin may result in an increase in mortality in patients with only atrial fibrillation
Mortality in patients with atrial fibrillation and heart failure (9 meta-analyses)	HR 1.14 (1.12 to 1.16)	⨁⨁⨁◯ Moderate	Digoxin likely results in an increase in mortality in patients with atrial fibrillation and heart failure

^a^Critically low/low quality in 5 out of 8 studies included

**Fig. 2:**
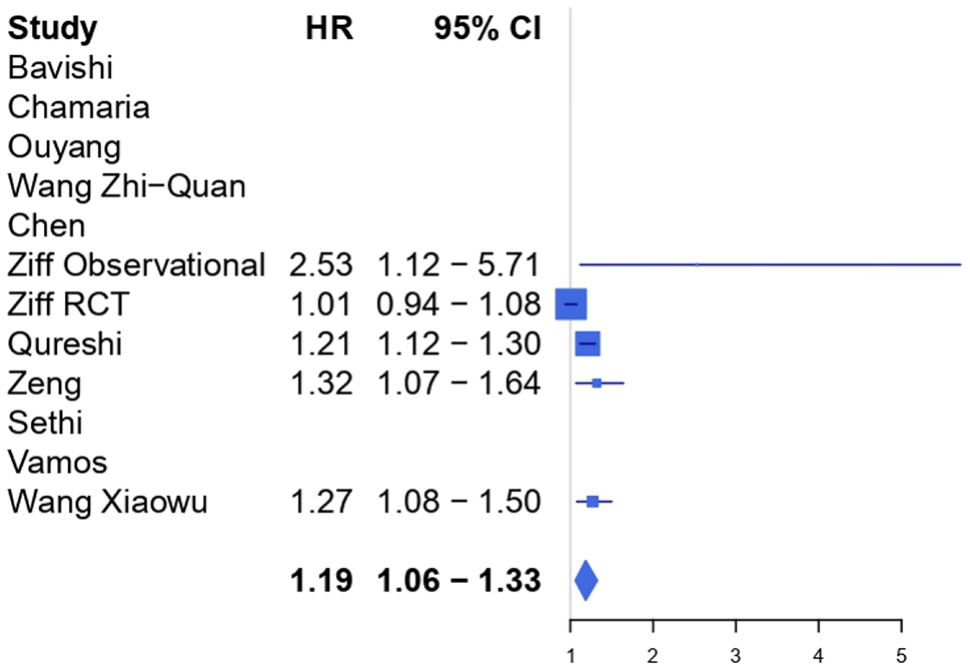
Forest plot for cardiovascular mortality

Data on cardiovascular mortality were provided by 5 studies (Fig. 2). Overall, the evidence suggests that digoxin might result in an increase in cardiovascular mortality (HR 1.19, 95%CI 1.06–1.33) with moderate certainty of evidence according to the GRADE (Table 4) and moderate heterogeneity (*I*^2^ 70.5%).

### Subgroup analysis according to the HF

As far as it concerns mortality in AF-only population (Fig. 1B), only 8 papers provided data concerning this outcome. Our analysis shows that digoxin may result in an increase in mortality in this group of patients (HR 1.23, 95%CI 1.19–1.28) with low certainty of evidence according to the GRADE (Table 4) and moderate heterogeneity (*I*^2^ 70.8%).

Mortality in patients affected by AF and HF outcome (Fig. 1C) was explored by 8 papers and provided similar results. Even in this population, digoxin was associated with an increase in mortality (HR 1.14, 95%CI 1.12–1.16) with moderate certainty of evidence according to the GRADE (Table 4) and no heterogeneity (*I*^2^ 0%).

## Discussion

Results from this umbrella review of meta-analyses indicate that the use of digoxin may be associated with an increased risk of all-cause and cardiovascular mortality in patients with AF.

The need for this umbrella review and meta-analysis came from literature analysis in which a growing number of observational studies reported a potential harmful effect of digoxin in AF patients [15, 16]. However, this evidence became conflicting after the publication of some meta-analyses providing discordant results. For this reason, we adopted the methodology of the umbrella review, which represents one of the highest levels of evidence synthesis currently available [17], to provide more robust data on the association between digoxin and mortality in patients with AF, given the lack of data from a controlled randomized setting. Our analysis indicates an association of digoxin use with all-cause and cardiovascular mortality in patients with AF.

Notably, the association with all-cause mortality persisted in the subgroup of patients with AF and HF, even if with a lower strength of association. The use of digoxin in AF patients with HF is well established in clinical practice, but it should be noted that consolidated evidence showed that the effect of digoxin may not be so evident when a stable haemodynamic has been already reached with other drugs such as diuretics and vasodilators [18], and that digoxin may work less when an activation of sympathetic system is present (e.g., acute decompensated HF) [19]. Thus, HF should not represent per se an indication to the use of digoxin as possible harmful effects are also evident in this subgroup of patients. One of the arguments for the still wide use of digoxin is its ability to reduce the rate of hospitalization and the improvement of symptoms in patients treated with this drug [20]. However, more recently, the TREAT-AF trial included patients with permanent AF and HF randomized to receive digoxin or bisoprolol [21]. This study showed no difference between the two treatments group regarding symptoms after 6 months of therapy [21].

Strengths of the study are that it is the first systematic umbrella review of evidence from meta-analyses including a large sample of patients; even if a number of patients may be counted more than once given the design of umbrella review, it still remains the largest number of subjects considered to our knowledge. Furthermore, we also performed an accurate quality evaluation, certainty of evidence analysis, and risk of bias.

The different results obtained from the present umbrella review and meta-analysis in comparison to other previously published meta-analyses may rely on several reasons including different selection of studies, definition of outcomes variable length of follow-up, and lack of quality evaluation of evidence. In addition, 3 previous meta-analyses had critically low quality, and 2 had low quality at AMSTAR evaluation.

Clinical implications of our results are relevant considering that a high number of patients are currently treated with digoxin worldwide. Clinicians should be aware that digoxin may be harmful in AF patients, especially in some specific settings such as in chronic kidney disease or electrolyte imbalance, both conditions increasing the risk of adverse effects. In addition, patients prescribed on digoxin should be adequately informed about the potential side effects and the need of regular medical and laboratory follow-up while taking this medication.

Our results indicate that digoxin should be considered only in patients who do not achieve an adequate rate control or who experience symptoms with other anti-arrhythmic drugs. In addition, digoxin may be considered in patients with contraindication to the use of beta blockers (e.g., pulmonary disease) or to the use of calcium channel antagonists (such as heart failure). The use of laboratory monitoring and careful electrocardiographic examination may help recognize the early signs of digoxin toxicity, allowing a prompt intervention to reduce the risk of mortality associated with supra-therapeutic values of plasma digoxin. Indeed, values exceeding the therapeutic range may result in an increased risk of pro-thrombotic [22] and pro-arrhythmogenic effect and in an increased endothelial platelet activation [19, 22, 23], all mechanisms leading to an increased risk of cardiovascular mortality.

There are limitations of this analysis to acknowledge. First, despite the umbrella review approach provides robust evidence regarding the association between digoxin and mortality, the inclusion of observational studies carries some intrinsic limitations, mainly due to the impossibility of eliminating the bias by indication, which implicates that patients prescribed on digoxin may be sicker than those not treated with this drug despite the multivariable adjustment for the most common comorbidities [24]. However, it should be noted that subgroup analysis of propensity-matched populations provided similar results [11]. However, what cannot be deduced from clinical studies is the reason for mortality, so we do not know if toxicity, arrhythmia, and HF were the causes of death. Indeed, data on serum digoxin concentration, renal function, acute coronary syndrome, potassium levels may provide important additional information to understand the association of digoxin with clinical outcomes. Furthermore, we do not know if patients were adequately followed after digoxin prescription.

In conclusion, despite its wide use, the use of digoxin should be considered with caution in patients with AF and should be reserved to those patients in whom an adequate rate control is difficult to achieve with other anti-arrhythmic drugs.

## Supplementary Information

Below is the link to the electronic supplementary material.

Supplementary file1 (DOCX 278 KB)
