# Supplementary material for: Effect of digoxin on all-cause and cardiovascular mortality in patients with atrial fibrillation with and without heart failure: an umbrella review of systematic reviews and 12 meta-analyses
Source: Eur J Clin Pharmacol. 2023 Mar 6;79(4):473–83. doi: 10.1007/s00228-023-03470-y (PMC10039090; doi:10.1007/s00228-023-03470-y)
Supplement: Supplementary file 1 — Supplementary file1 (DOCX 278 KB) [file 228_2023_3470_MOESM1_ESM.docx]

**Effect of digoxin on all-cause and cardiovascular mortality in patients with atrial fibrillation with and without heart failure: an umbrella review of systematic reviews and 12 meta-analyses**

Gianluca Gazzaniga MD(1), Danilo Menichelli MD(2), Francesco Scaglione MD, PhD(3,4), Alessio Farcomeni PhD(5), Arianna Pani MD(3), Daniele Pastori, MD, PhD(6)

Affiliations

1. Department of Medical Biotechnology and Translational Medicine, Postgraduate School of Clinical Pharmacology and Toxicology, Università degli Studi di Milano, 20122, Milan, Italy.
2. Department of General and Specialized Surgery "Paride Stefanini", Sapienza University of Rome, 00185 Rome, Italy.
3. Department of Oncology and Hemato-Oncology, Università degli Studi di Milano, 20122 Milan, Italy.
4. Department of Chemical-Clinical and Microbiological Analyses, Grande Ospedale Metropolitano Niguarda, 20162 Milan, Italy.
5. Department of Economics and Finance, University of Rome "Tor Vergata", 00133 Rome, Italy.
6. Department of Clinical, Internal, Anesthesiological, and Cardiovascular Sciences, Sapienza University of Rome, 00185 Rome, Italy.

**Correspondence to** Prof. Daniele Pastori. Department of Clinical, Internal Medicine, Anesthesiological and Cardiovascular Sciences, “Sapienza” University of Rome, Viale del Policlinico 155, 00161, Rome, Italy. E-mail: daniele.pastori@uniroma1.it. Tel. +390649970941 Fax +390649972309.

**Supplementary Material 1. Search strategy**

**Search Strings 19/10/2021**

*Embase: 183 papers*

(digoxin OR 'digitalis') AND 'atrial fibrillation' AND ('meta analysis'/de OR 'meta analysis topic'/de OR 'systematic review'/de) AND 'review'/it

*Pubmed: 45 papers*

(digoxin OR digitalis) AND atrial fibrillation (Filters applied: Meta-Analysis, Systematic Review)

*Web of Science: 169 papers*

((ALL=(digoxin)) OR ALL=(digitalis)) AND ALL=(atrial fibrillation) and Review Articles (Document Types)

Total: 397 (350 with no duplicates)

**Supplementary Figure 1. PRISMA 2020 flow diagram for new systematic reviews which included searches of databases, registers and other sources.**

**Supplementary Figure 2. Funnel plots: all-cause mortality in all patients (Panel A), cardiovascular mortality in all patients (Panel B), all-cause mortality in patients with only atrial fibrillation (Panel C) and patients with atrial fibrillation and heart failure (Panel D),**

**A**

**B**

**C**

**D**
